# An automated 3D-printed perfusion bioreactor combinable with pulsed electromagnetic field stimulators for bone tissue investigations

**DOI:** 10.1038/s41598-022-18075-1

**Published:** 2022-08-16

**Authors:** Stefano Gabetti, Beatrice Masante, Andrea Cochis, Giovanni Putame, Alessandro Sanginario, Ileana Armando, Elisa Fiume, Alessandro Calogero Scalia, Farah Daou, Francesco Baino, Simona Salati, Umberto Morbiducci, Lia Rimondini, Cristina Bignardi, Diana Massai

**Affiliations:** 1 PolitoBIOMed Lab, Department of Mechanical and Aerospace Engineering, Politecnico di Torino, Turin, Italy; 2 Interuniversity Center for the Promotion of the 3Rs Principles in Teaching and Research, Turin, Italy; 3 Laboratory of Biomedical Materials, Center for Translational Research on Autoimmune and Allergic Disease-CAAD, Department of Health Sciences, University of Piemonte Orientale UPO, Novara, Italy; 4 Department of Electronics and Telecommunications, Politecnico di Torino, Turin, Italy; 5 Department of Information Engineering, University of Brescia, Brescia, Italy; 6 Department of Applied Science and Technology, Politecnico di Torino, Turin, Italy; 7 IGEA Clinical Biophysics, Carpi, Italy

**Keywords:** Biomedical engineering, Tissue engineering

## Abstract

In bone tissue engineering research, bioreactors designed for replicating the main features of the complex native environment represent powerful investigation tools. Moreover, when equipped with automation, their use allows reducing user intervention and dependence, increasing reproducibility and the overall quality of the culture process. In this study, an automated uni-/bi-directional perfusion bioreactor combinable with pulsed electromagnetic field (PEMF) stimulation for culturing 3D bone tissue models is proposed. A user-friendly control unit automates the perfusion, minimizing the user dependency. Computational fluid dynamics simulations supported the culture chamber design and allowed the estimation of the shear stress values within the construct. Electromagnetic field simulations demonstrated that, in case of combination with a PEMF stimulator, the construct can be exposed to uniform magnetic fields. Preliminary biological tests on 3D bone tissue models showed that perfusion promotes the release of the early differentiation marker alkaline phosphatase. The histological analysis confirmed that perfusion favors cells to deposit more extracellular matrix (ECM) with respect to the static culture and revealed that bi-directional perfusion better promotes ECM deposition across the construct with respect to uni-directional perfusion. Lastly, the Real-time PCR results of 3D bone tissue models cultured under bi-directional perfusion without and with PEMF stimulation revealed that the only perfusion induced a ~ 40-fold up-regulation of the expression of the osteogenic gene collagen type I with respect to the static control, while a ~ 80-fold up-regulation was measured when perfusion was combined with PEMF stimulation, indicating a positive synergic pro-osteogenic effect of combined physical stimulations.

## Introduction

Nowadays, due to the population ageing coupled with rising of obesity and decreased physical activities, bone fractures and their clinical management represent a heavy socio-economic burden^1,2^ complemented by a dramatically growing need for bone replacement worldwide^3,4^.

In fact, although bone can usually regain functionality by self-healing, there are pathological conditions such as nonunion or large bone defects due to trauma, infections, tumors or osteoporosis in which self-healing fails, causing severe pain and immobility to patients^5,6^. Besides the conventional surgical procedures adopted for managing critical-sized bone defects, bone tissue engineering (BTE) is emerging as a promising strategy for generating in vitro functional bone tissue substitutes to be implanted for promoting in vivo bone regeneration^7^. BTE approaches are based on the effective interplay among osteogenic cells, three-dimensional (3D) porous scaffolds, and physiological chemical and physical stimuli^8,9^. Currently, a direct translation of BTE strategies to clinical use still remains challenging due to scientific, technical, and regulatory limitations^10–12^, and BTE substitutes are mostly adopted as 3D bone tissue models for in vitro bone research and pre-clinical studies^13,14^. For example, it has been demonstrated that mechanical forces, such as compression and fluid flow-induced shear stress, influence or even drive stem cells differentiation into mature bone lineages^15–17^. Therefore, for a clear understanding of the mechanotransduction mechanisms driving bone tissue development, homeostasis, and regeneration, the in vitro modelling and analysis of 3D bone tissue models exposed to controlled native-like physical stimuli would be essential^18^.

In this context, several bioreactors have been developed and adopted as powerful investigation tools for providing in vitro defined native-like physical stimuli^19–27^. Technically, bioreactors imposing hydrostatic pressure were developed to mimic the native-like compression^28–31^. Along with this approach, a variety of studies showed that direct perfusion, by guaranteeing continuous medium flow through the 3D cultured constructs, ensures efficient mass transport during both cell seeding and tissue culture^32,33^ and exposes the constructs to fluid flow-induced shear stress profiles that can promote proliferation and differentiation of osteoblasts and foster bone mineralization^31,34–36^. For example, Bancroft and colleagues developed a direct uni-directional perfusion bioreactor (total medium volume = 200 mL) based on a peristaltic pump and observed increased deposition of mineral extracellular matrix (ECM) produced by marrow stromal osteoblasts seeded on titanium fiber mesh scaffolds, when cultured under perfusion for 16 days^34,37^. Moreover, adopting the same setup and constructs, the combination of uni-directional perfusion and osteogenic medium resulted in enhanced proliferation and differentiation of mesenchymal stem cells^38^. Subsequently, the bi-directional/oscillating perfusion mode, which was at first introduced to improve cell seeding efficiency^39–42^, has emerged as an effective strategy to stimulate the constructs more uniformly by better recapitulating the multi-directional movement of the interstitial fluid within the native bone^43,44^, promoting osteogenic differentiation as well^45–47^. In 2018, Beşkardeş et al. developed a bi-directional perfusion bioreactor based on a syringe pump and found that bi-directional perfusion, combined with osteogenic culture medium, enhanced osteogenic differentiation of pre-osteoblasts seeded on chitosan-hydroxyapatite scaffolds after 21 days of culture^47^. However, only one study compared the effects of uni- and bi-directional perfusion on 3D bone constructs, demonstrating that after 6 days bi-directional flow promoted more uniform cell proliferation and increased early osteogenic effects with respect to uni-directional flow^48^. This result was obtained by using two different non-automated perfusion systems: a uni-directional perfusion bioreactor (total culture medium volume = 250 mL) based on a pump for chromatography, and a bi-directional perfusion device based on a syringe pump acting on a flexible membrane (total culture medium volume = 1.5 mL). Moreover, due to their peculiar architectures and to the lack of automated control, the two perfusion devices needed different manual operating procedures. For overcoming the intrinsic limitations of manual procedures, a further crucial feature in advanced bioreactors is automation, which allows enhancing environmental control and reducing user intervention, thus increasing process reproducibility and standardization. In 2018, Schmid and co-workers developed a perfusion bioreactor with automated cell seeding and active control of oxygen concentration during the culture, facilitating the investigation of BTE constructs with high homogeneity and viability^33^.

Besides the well-known mechanical stimuli characterizing the bone tissue environment in vivo, in the last decades further physical stimuli, clinically applied for boosting bone tissue regeneration, have been investigated. In particular, the non-invasive pulsed electromagnetic field (PEMF) stimulation was demonstrated to foster bone cell proliferation, differentiation, and ECM protein expression, with evident beneficial effects in promoting endogenous bone healing^49–52^. PEMF stimulation induces a secondary electric field in the exposed tissue, like the one generated in native bone during the transduction of mechanical energy into electrical energy (bone piezoelectric behavior)^53^, which can trigger the cell membrane depolarization and consequently stimulate ion currents^54^. However, due to the variety of PEMF stimulators and setups adopted, a complete understanding of the biological mechanisms induced by PEMF is yet missing and PEMF stimulation is empirically applied in the orthopedic clinical practice^53,55,56^. Thus, new investigation tools and approaches are required for performing in-depth studies that could lead to define optimal standardized PEMF protocols for treating the different pathological conditions.

Inspired by this scenario, we developed a novel automated perfusion bioreactor that allows culturing 3D constructs under tunable, automated uni- or bi-directional perfusion and that can be combined with PEMF stimulators. Computational modelling supported the design optimization of the bioreactor culture chamber, allowing characterizing the fluid dynamics and the magnetic field across it. Rapid, flexible and cost-effective 3D-printing techniques were adopted for the manufacturing phase. A user-friendly control unit was appositely developed for enabling setting and automated control of the perfusion unit, with the final aim of reducing the user dependency and increasing process reproducibility. For assessing the bioreactor performances in terms of perfusion, preliminary biological tests were performed on 3D bone tissue models, obtained by seeding human mesenchymal stem cells (hMSCs) on commercial bone substitutes, cultured under uni- or bi-directional perfusion. The biological effects of the different imposed culture conditions were evaluated in terms of cell viability, release of the early osteogenic differentiation marker alkaline phosphatase (ALP), and ECM deposition. Lastly, to verify the performances of the combined platform and to investigate the potential pro-osteogenic effect of combining bi-directional perfusion and PEMF stimulation, a real-time PCR-based test was performed culturing 3D bone tissue models for 14 days under three defined conditions: static condition, bi-directional perfusion, and bi-directional perfusion combined with PEMF stimulation. The expression of the osteogenic genes ALP and collagen type I were evaluated at the end of the culture by Real-time PCR.

## Materials and methods

### Bioreactor design, components and working principle

**Figure 1:**
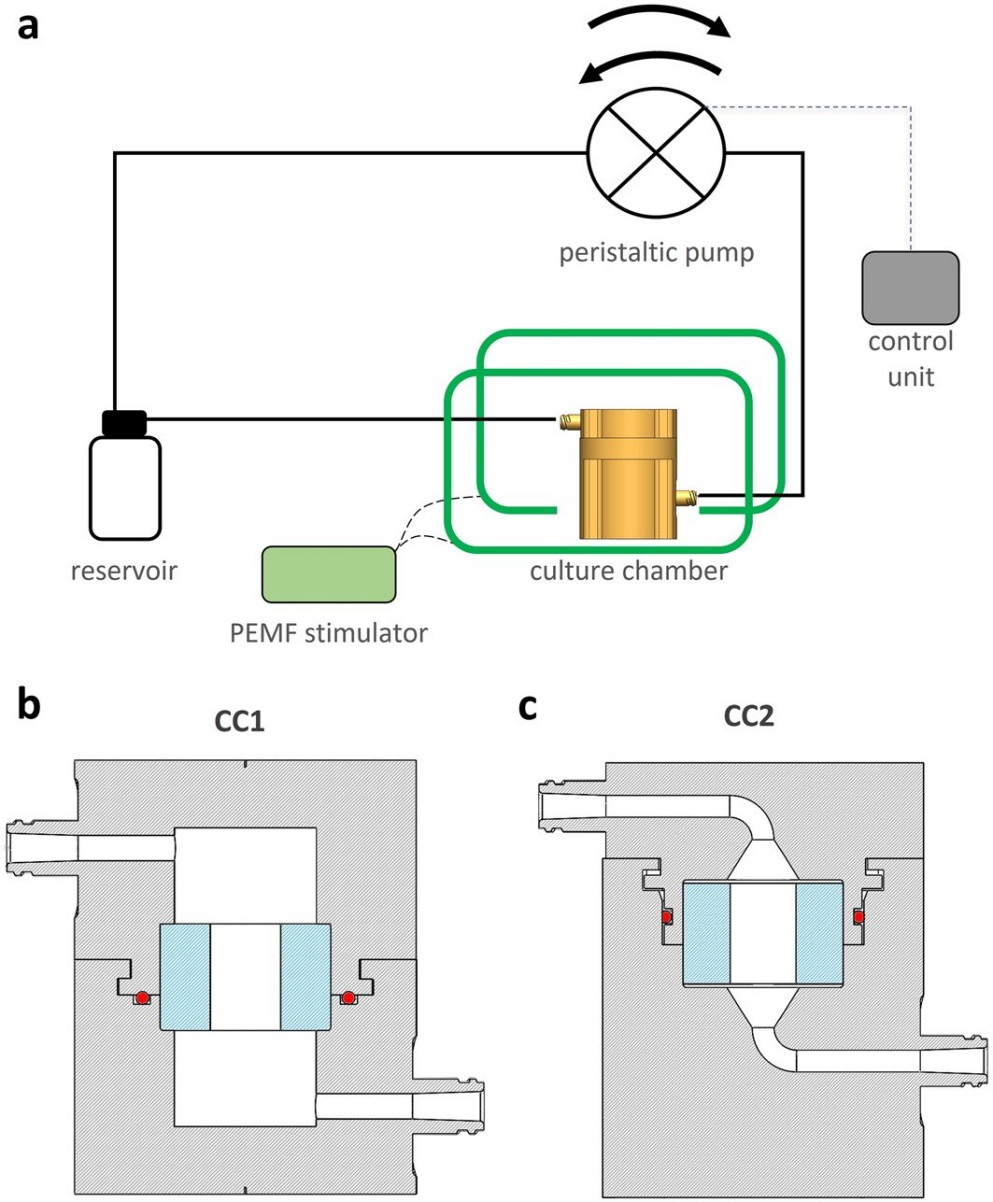
Bioreactor scheme and culture chamber design. (**a**) Schematic drawing of the bioreactor setup combined with the PEMF stimulator, with the connections among the culture chamber, the perfusion unit, and the control unit. Section views of the 3D models of CC1 (**b**) and CC2 (**c**), in grey the culture chamber, in blue the silicone holder, in red the o-ring.

The proposed bioreactor was designed for providing, in a controlled manner, tunable direct perfusion and to be combinable with a PEMF stimulator. In detail, the bioreactor is composed of: (1) a culture chamber, for housing the cultured 3D construct; (2) a perfusion unit, for providing uni- or bi-directional perfusion; (3) a control unit, for setting and automatically controlling the perfusion unit from outside the incubator. The bioreactor is combinable with a PEMF stimulation device (Fig. 1a) to deliver individual or combined physical stimulations (uni- or bi-directional perfusion and/or PEMF stimulation) to the cultured constructs.

**Figure 2:**
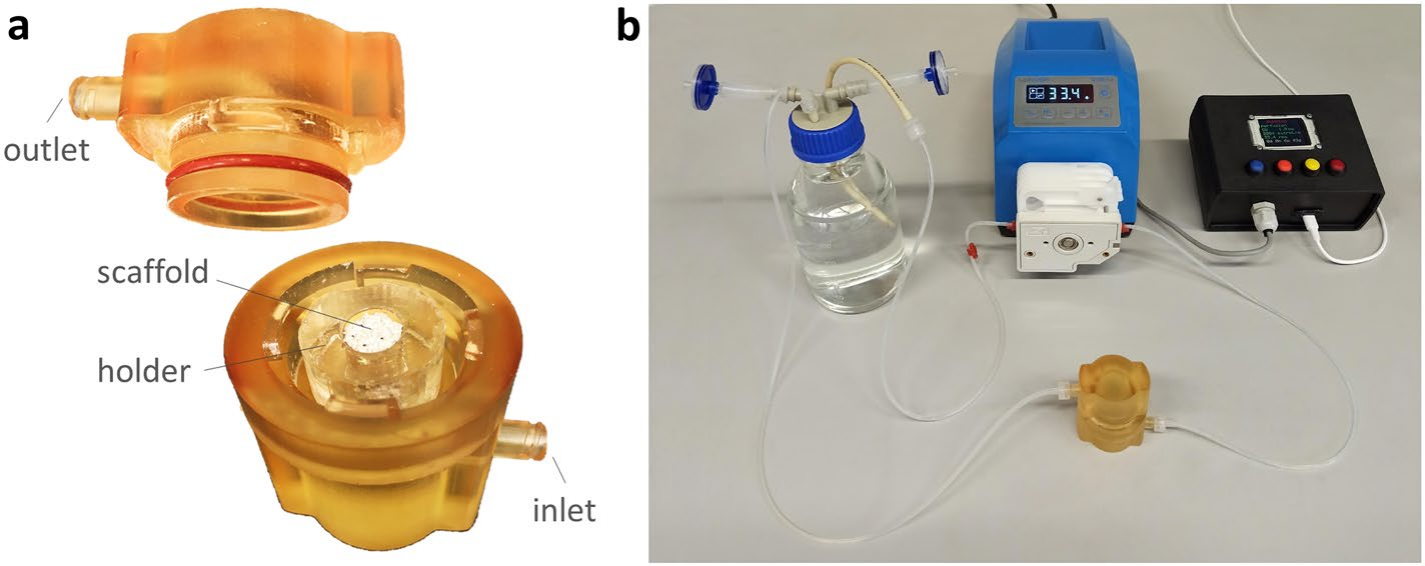
Bioreactor culture chamber. (**a**) The 3D printed CC2 is composed of a top part, equipped with the o-ring and the flow outlet, and a bottom part, housing the silicone holder and an exemplary scaffold. (**b**) Picture of the bioreactor components: culture chamber, perfusion unit, and control unit.

As regards the bioreactor development, supported by computational fluid dynamics (CFD) simulations, two versions of the culture chamber (identified as “CC1” and “CC2”) were designed (SOLIDWORKS, Dassault Systèmes, France). Both chamber layouts consist of two cylindrical screwable parts, equipped with inlet and outlet channels and luer threads (Fig. 1b,c). Tailored flexible cylindrical holders, to be press-fit inserted within the culture chamber, allow to house 3D cylindrical constructs of different size [diameter (d) = 7–10 mm; height (h) = 1–15 mm]. CC1 is characterized by an external (d = 48 mm, h = 61 mm) and internal (d = 20 mm, h = 42 mm) cylindrical geometry (culture chamber working volume = 10 mL), the inlet and outlet channels connect laterally to the internal volume, and watertightness is achieved by combining an interlocking mechanism and an O-ring inserted in the bottom part of the chamber for axial sealing (Fig. 1b). CC2 layout was designed to improve the flow field distribution within the chamber and to reduce the working volume. In detail, CC2 has an external cylindrical geometry (d = 48 mm, h = 65 mm), while internally it is characterized by a truncated cone geometry upstream and downstream of the cylindrical central part (d = 24 mm, h = 15 mm), with a working volume of 2.5 mL. The inlet and outlet channels connect co-axially to the internal volume due to curved paths, and an O-ring is located on the top part of the chamber for radial sealing (Fig. 1c). Based on CFD outcomes, CC2 was selected as the optimal layout that was then 3D-printed by stereolithography (SLA) using the biocompatible Dental SG Resin (Form 3, Formlabs, United States), setting a layer thickness of 50 μm (Fig. 2a). Four cylindrical holders (internal d = 7, 8, 9, or 10 mm; external d = 24 mm; h = 15 mm) were manufactured by casting biocompatible silicone (SYLGARD 184, Dow Corning, United States) into modular acrylonitrile butadiene styrene (ABS) molds, 3D-printed by fused deposition modeling (uPrint SE Plus, Stratasys, United States), and curing them at 60 °C for 5 h.

The culture chamber is connected to the perfusion unit (total culture medium volume = 50 mL), which is composed of: a culture medium reservoir with inlet and outlet ports, a medium sampling port, and air filters; oxygen permeable platinum-cured silicone tubing (Darwin Microfluidics, France); luer fittings (IDEX Health & Science, United States); and a peristaltic pump (G100-1 J, Longer Precision Pump, China; for flow rate range see Supplementary Table S1) suitable to be incubated and to be connected to and controlled by the control unit (Fig. 2b).

The control unit, connected to the pump via RS-485 serial communication, is enclosed in a compact box (135 × 130 × 60 mm^3^) and equipped with a microcontroller board (Arduino Micro, Arduino, Italy) that runs a purpose-built software. A user-friendly interface, based on four push buttons and one LCD display (Arduino, Italy), allows setting the perfusion parameters (Supplementary Table S1, Fig. 2b). In uni-directional mode, the flow direction can be set by selecting the direction of rotation of the pump head; however, in order to promote the outflow of possible air bubbles, a bottom-to-top flow is recommended. In bi-directional mode, the user can set the cycle duration, i.e., the time interval after which the flow direction is automatically inverted. Preliminary tests for assessing the bioreactor performance in terms of watertightness and reliability were performed (see Supplementary Material).

To deliver PEMF stimulation to the cultured constructs, a commercial PEMF stimulator composed of a generator and two solenoids was selected (magnetic field intensity = 1.5 mT, frequency = 75 Hz, IGEA Clinical Biophysics, Italy) and the bioreactor culture chamber was placed between the solenoids.

### CFD simulations and wall shear stress estimation

For supporting the optimization of the culture chamber design, CFD simulations were performed. In detail, the 3D geometries of CC1 and CC2 were discretized with 3.41 × 10^6^ and 4.99 × 10^5^ elements, respectively, using tetrahedral elements for the bulk and hexahedral elements for the boundary layer (COMSOL Multiphysics 5.3, COMSOL Inc., Sweden). The 3D construct, assumed as a reference cylinder (diameter = 10 mm, height = 15 mm), was modelled as a homogeneous and isotropic porous medium, imposing the properties (permeability *k* = 3 × 10^–10^ m^2^, porosity *φ* = 60%^57^) of the commercial Bio-Oss scaffold (Geistlich Pharma AG, Switzerland) adopted for the preliminary biological tests. The culture medium was modelled as an incompressible, Newtonian fluid (density *ρ* = 9.94 × 10^2^ kg/m^3^, dynamic viscosity *µ* = 6.89 × 10^–4^ Pa s at 37 °C). Using a finite element-based commercial code (COMSOL Multiphysics 5.3, COMSOL Inc., Sweden), the governing equations of fluid motion were solved in their discretized form in the fluid domain, while the Brinkman equation^58^ in its discretized form was adopted for describing perfusion in the porous domain. Four steady-state simulations were performed for each culture chamber layout, with and without the 3D construct and prescribing uni-directional perfusion with flow rate values of 0.3 and 1.0 mL/min (imposing a parabolic velocity profile) at the inlet of the culture chamber bottom. A reference pressure was imposed at the outlet, and the no-slip condition was applied at the internal walls of the culture chambers. Moreover, in order to investigate the development of the flow upstream of the construct within CC1 and CC2, the velocity field distributions were analyzed at 3 different horizontal sections of the culture chamber bottom (see Supplementary Fig. S1).

The simulated flow regimes within CC1 and CC2, expressed in terms of Reynolds number (Re) calculated considering the internal diameter of the inlet channel (3.7 mm) as the characteristic length, resulted to be laminar (Re = 2.48 for 0.3 mL/min; Re = 8.28 for 1.0 mL/min). Following the adoption of the Brinkman equation, the wall shear stress (*τ*_*w*_) in the porous construct was evaluated using the expression obtained by Wang and Tarbell^59,60^, which provides an accurate estimation for constructs with permeability higher than 10^–10^ m^2^:

$${\tau }_{w}=\frac{4}{\pi }\frac{\mu }{\sqrt{k}}{v}_{avg}$$

where *µ* is the dynamic viscosity of the culture medium, *v*_*avg*_ is the average velocity of the culture medium within the construct as obtained from CFD simulations, *k* is the construct permeability, and a null cell density was assumed.

### Electromagnetic field simulations

Electromagnetic field simulations were performed to assess the suitability of the proposed bioreactor to be used in combination with a reference PEMF stimulator^61^. In detail, a 3D steady-state simulation was carried out adopting a finite element-based commercial code (COMSOL Multiphysics 5.3, COMSOL Inc., Sweden) for investigating the distribution of the magnetic field within CC2 when placed between the two solenoids of the PEMF stimulator (Supplementary Fig. S2). The CC2 internal geometry was discretized using 2.25 × 10^6^ tetrahedral and 1.61 × 10^5^ triangular elements. The Ampere’s Law was solved in its discretized form imposing a current of 160 mA at each solenoid. A magnetic insulation condition (n × A = 0, where A is the magnetic potential) was prescribed at the domain boundaries. Each component was modelled according to its electromagnetic properties (Supplementary Table S2).

### 3D bone tissue model preparation and culture under perfusion

Commercially available bone marrow-derived human mesenchymal stem cells (hMSCs) were obtained from Merck (C-12974 PromoCell GmbH, Germany) and cultivated in low-glucose Dulbecco’s modified Eagle Medium (DMEM, Sigma-Aldrich, United States) supplemented with 15% fetal bovine serum (FBS, Sigma-Aldrich, United States) and 1% penicillin/streptomycin into a standard incubator (37 °C, 5% CO_2_). Each 3D bone tissue model was obtained by seeding 4 × 10^6^ cells into a pre-molded cylinder (diameter = 10 mm, height = 6 mm) of commercial bone substitute Bio-Oss (Geistlich Pharma AG, Switzerland), as previously described^62^. The 3D constructs were then statically pre-cultured in incubator for 48 h to allow full cell adhesion and spread.

For perfusion culture, the 3D bone tissue model was inserted in the flexible holder and housed in the bioreactor culture chamber, previously filled with 1 mL of fresh culture medium. The bioreactor was then located in incubator, the control unit was set (flow rate = 0.3 mL/min, uni- or bi-directional (cycle duration = 2 h) perfusion mode), and the pump was automatically put into operation (Supplementary Fig. S3a). The construct was dynamically cultured for 3 or 6 days, under continuous perfusion, with a total culture medium volume of 50 mL. Analogous constructs were cultured in static conditions for the same time intervals as control tests (Supplementary Fig. S4), with a total culture medium volume of 3 mL that was changed every 3 days, following the physiological degradations of the culture medium key components, such as serum. Experiments were performed in triplicate.

### Assessments of 3D bone tissue models cultured under perfusion

At day 3 or day 6 time-points, the effect of the applied uni- or bi-directional perfusion was investigated in terms of cell viability and alkaline phosphatase (ALP) release. Cell viability was evaluated by assessing cell metabolic activity by the colorimetric Alamar blue assay (Life Technologies, Italy). At each time-point, constructs cultured under dynamic or static conditions were collected, then submerged by the Alamar blue solution and incubated for 4 h in the dark. Afterwards, 100 µL were moved into a black 96-well plate, and the fluorescence signals were evaluated with a spectrophotometer (Spark, Tecan Trading AG, Switzerland) using a 590 nm wavelength for the reading. The early osteogenic marker ALP released by constructs cultured under dynamic or static conditions was measured in the supernatants using a colorimetric assay (ab83369 from AbCam, United Kingdom). Briefly, 80 µL of each supernatant was collected and mixed with 50 µL of the pNPP solution and 10 µL of the ALP enzyme. After 60 min, the optical density was evaluated by spectrophotometry (Spark, Tecan Trading AG, Switzerland) using a 405 nm wavelength. Moreover, at day 6, the deposition of ECM was verified by histology. The 3D bone tissue models cultured under dynamic or static conditions were collected, fixed with 10% formalin, dehydrated by the alcohols' scale (70–90–100), and embedded in resin (Tecnovit 7200, Kulzer, Germany). Afterwards, the constructs were horizontally (top-down) sliced in parallel to the flow direction, along the mechanically polished diameter and surface, to a final thickness of 80 μm. Histological analysis was performed on the central slices representative for the core of the scaffolds by means of Toluidine blue. Images were acquired using an optical digital scanner (NanoZoomer S60, Hamamatsu, Japan).

### Combined bi-directional perfusion and PEMF stimulation culture and Real-time PCR analysis

To verify the performances of the combined platform and to investigate the biological effect of combining bi-directional perfusion and PEMF stimulation, 3D bone tissue models, prepared as previously described, were cultured for 14 days under: (i) static culture (control); (ii) bi-directional perfusion (flow rate = 0.3 mL/min, cycle duration = 2 h); (iii) bi-directional perfusion (flow rate = 0.3 mL/min, cycle duration = 2 h) combined with PEMF stimulation (magnetic field intensity = 1.5 mT, frequency = 75 Hz, exposure time = 24 h/day, see Supplementary Fig. S3b). In order to avoid any interference due to biochemical stimulation, constructs were cultivated with maintenance medium (DMEM). At the end of the culture, Real-time PCR was performed to evaluate the expression of the early osteogenic gene ALP and of the late osteogenic gene collagen type I (COL1). Experiments were performed in triplicate. Total RNA extraction was performed using TRIzol (Invitrogen Life Technologies, Italy) according to the manufacturer’s instructions. Briefly, samples were placed in multiwell cell culture plates and incubated with 500 μL of TRIzol solution at room temperature for 10 min. The resulting solutions were transferred to 1.5 mL microtubes, and 100 μL of chloroform was added on ice and mixed well. The samples were kept at room temperature for 2–3 min, and then centrifuged at 12,000×*g* for 15 min at 4 °C. The upper aqueous layers were transferred to new 1.5 mL microtubes, and the same corresponding volume of isopropanol was added and mixed well. The samples were kept on ice for 10 min, and then centrifuged at 12,000×*g* for 10 min at 4 °C. The resulting pellets were then washed twice with 75% ethanol, by adding 500 μL of 75% ethanol to the pellets, vortexing to detach the pellet, centrifuging at 7500×*g* for 5 min at 4 °C, and then discarding the supernatant. The pellets were allowed to dry and then resuspended in 20 μL of diethylpyrocarbonate (DEPC)-treated water and stored at − 80 °C until use. The quantity and purity of the recovered RNA were determined via absorbance measurements at 230, 260, and 280 nm using the NanoPhotometer N60 Micro-Volume UV–Vis Spectrophotometer (Implen, United States). Gene expression analysis was performed using the two-step Real-time PCR. First, retrotranscription was performed to the RNA templates (0.2 μg) using the iScript gDNA Clear cDNA Synthesis Kit (Bio-Rad Laboratories, United States), according to the manufacturer’s instructions, and a thermal cycler (Mastercycler X50s, Eppendorf, Germany). The obtained cDNA templates were stored at − 20 °C until further use. Real-time PCR was performed using the SsoAdvanced Universal SYBR Green Supermix (Bio-Rad Laboratories, United States) and a thermal cycler (C1000 Touch Thermal Cycler, CFX96 Real-Time System, Bio-Rad Laboratories, United States). In brief, each reaction consisted of a total volume of 20 µL containing 1 µL of each primer, 2 µL of cDNA, 10 µL SYBR Green super mix and 6 µL of nuclease-free water. Each PCR reaction was run in technical triplicates. The thermal cycling conditions adopted were: 95 °C for 30 s, followed by 40 cycles of amplification at 95 °C for 5 s, and 60 °C for 15 s, and eventually the melting curves were analyzed. The target genes used were ALP and collagen type I alpha 1 chain (COL1A1), whereas, the reference gene used was the ribosomal protein L34. For data analysis, the fold change (FC) of each gene expression was calculated using the 2^–ΔΔCt^ method, and the reference gene was used to normalize the results.

### Statistical analysis

All biological experiments were performed in triplicate and results were statistically analyzed using the SPSS software (v.20.0, IBM, United States). Data normal distribution and homogeneity of variance were confirmed by the Shapiro–Wilk’s and the Levene's test, respectively; then, groups were compared by the one-way ANOVA using the Tukey's test as post-hoc analysis. Significant differences were established at p < 0.05.

## Results

### CFD simulations and wall shear stress estimation

**Figure 3:**
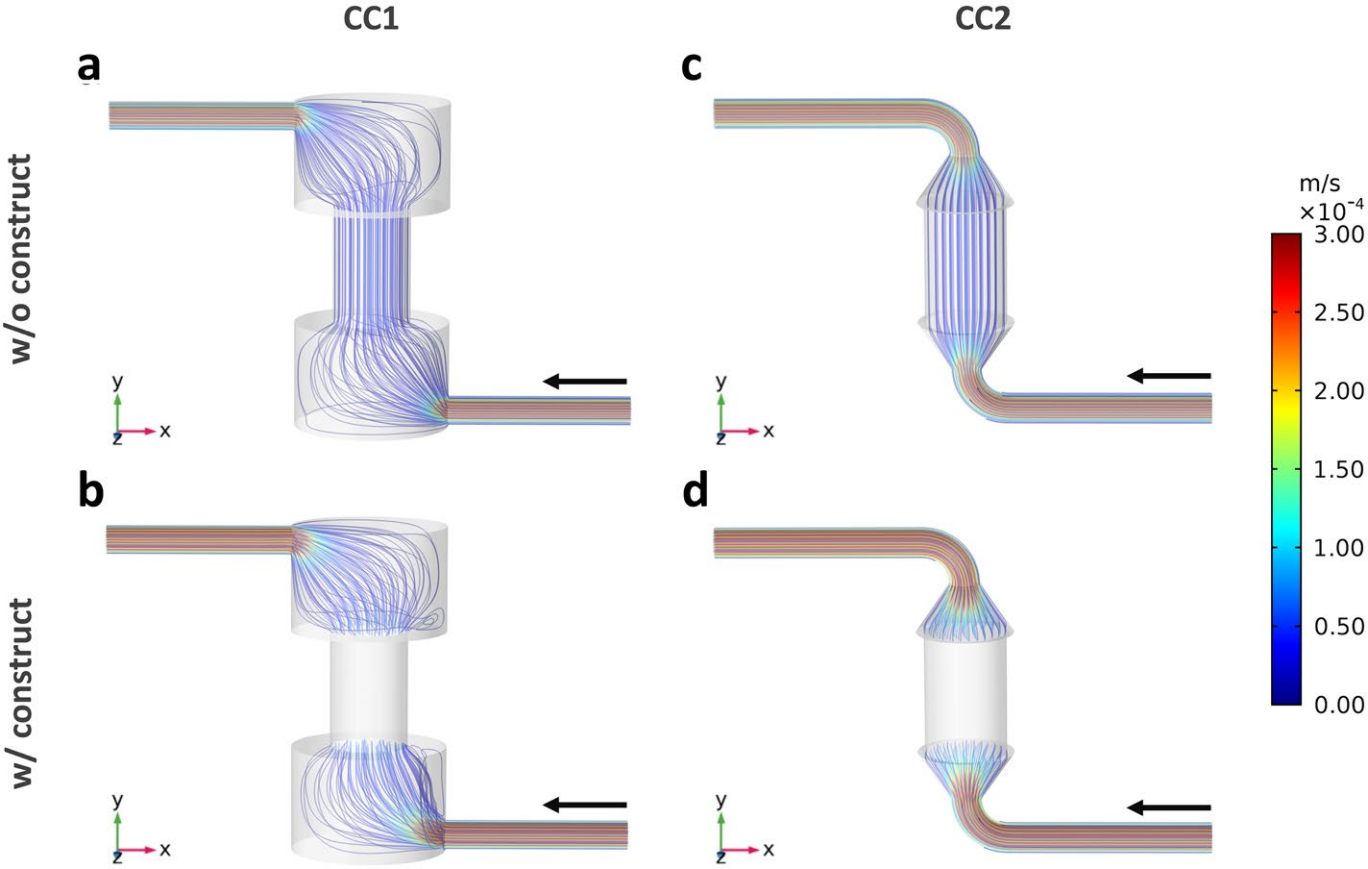
Flow streamlines developing within CC1 and CC2 imposing a modeled flow rate of 0.3 mL/min and color-coded compared with velocity values. (**a**) CC1 without construct. (**b**) CC1 with an inserted construct modelled as porous medium. (**c**) CC2 without construct. (**d**) CC2 with an inserted construct modelled as porous medium.

**Figure 4:**
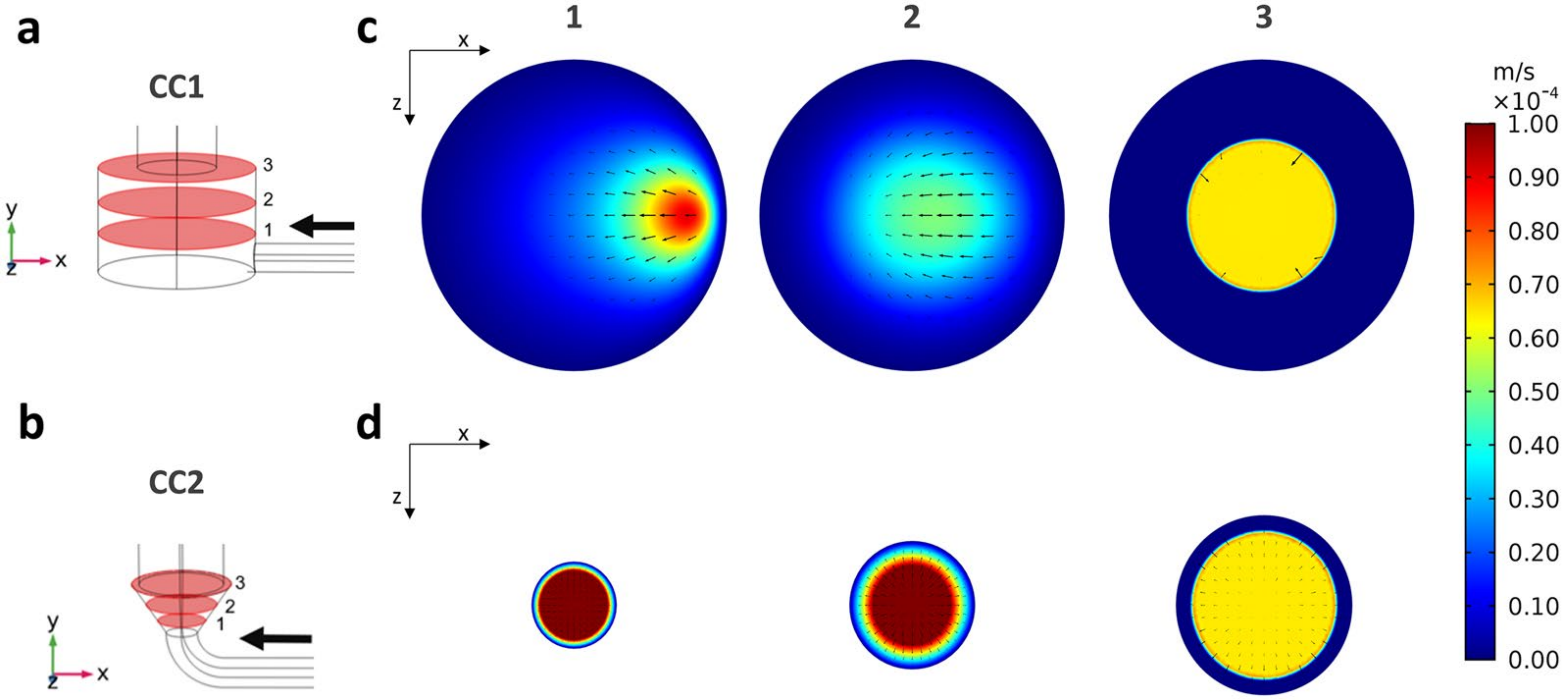
Development of the flow upstream of the construct within CC1 and CC2, analyzed at three different horizontal sections. (**a**) Bottom part of CC1 with horizontal sections. (**b**) Bottom part of CC2 with horizontal sections. Contour plots of the velocity component along the longitudinal axis of the culture chamber with vectors of in-plane velocity components for the three horizontal sections of CC1 (**c**) and of CC2 (**d**).

The CFD simulations allowed characterizing the hydrodynamics within CC1 and CC2 layouts at the imposed flow rates of 0.3 mL/min (Fig. 3) and 1 mL/min (Supplementary Fig. S5) for finally defining the optimal layout. In detail, the flow streamlines developing within CC1 reveal recirculation regions in the bottom and top parts of culture chamber, both without (Fig. 3a, Supplementary Fig. S5a) and with the construct inserted (Fig. 3b, Supplementary Fig. S5b). Differently, within CC2 the flow streamlines follow the internal geometry of the culture chamber avoiding recirculation regions, both without (Fig. 3c, Supplementary Fig. S5c) and with the construct inserted (Fig. 3d, Supplementary Fig. S5d). As regards the velocity field upstream of the construct (Fig. 4), the contour plots of the longitudinal velocity component show that the velocity field within CC1 is unevenly distributed (Fig. 4a), with the maximum velocity value misaligned with respect to the longitudinal axis close to the inlet and a flattened flow profile at the entrance of the construct. As regards the in-plane velocity vectors, they present a diverging pattern close to the inlet while a converging pattern approaching the construct (Fig. 4a). Differently, in CC2 the velocity profile is symmetric and parabolic everywhere upstream of the construct (Fig. 4b). Moreover, within CC1 it is possible to observe the presence of wide regions characterized by very low or null velocity where flow stagnation can occur, particularly at the bottom of the culture chamber (Fig. 4a), while in CC2 only the regions close to the walls are exposed to low or null velocities (Fig. 4b). Therefore, CC2 was selected as the optimal layout to be manufactured. The wall shear stress values within the construct, calculated from the average velocity values obtained from the CFD analysis, turn out to be 3.23 or 10.75 mPa for both CC1 and CC2, depending on the imposed flow rates (0.3 and 1.0 mL/min, respectively, see Supplementary Table S3).

### Electromagnetic field simulations

**Figure 5:**
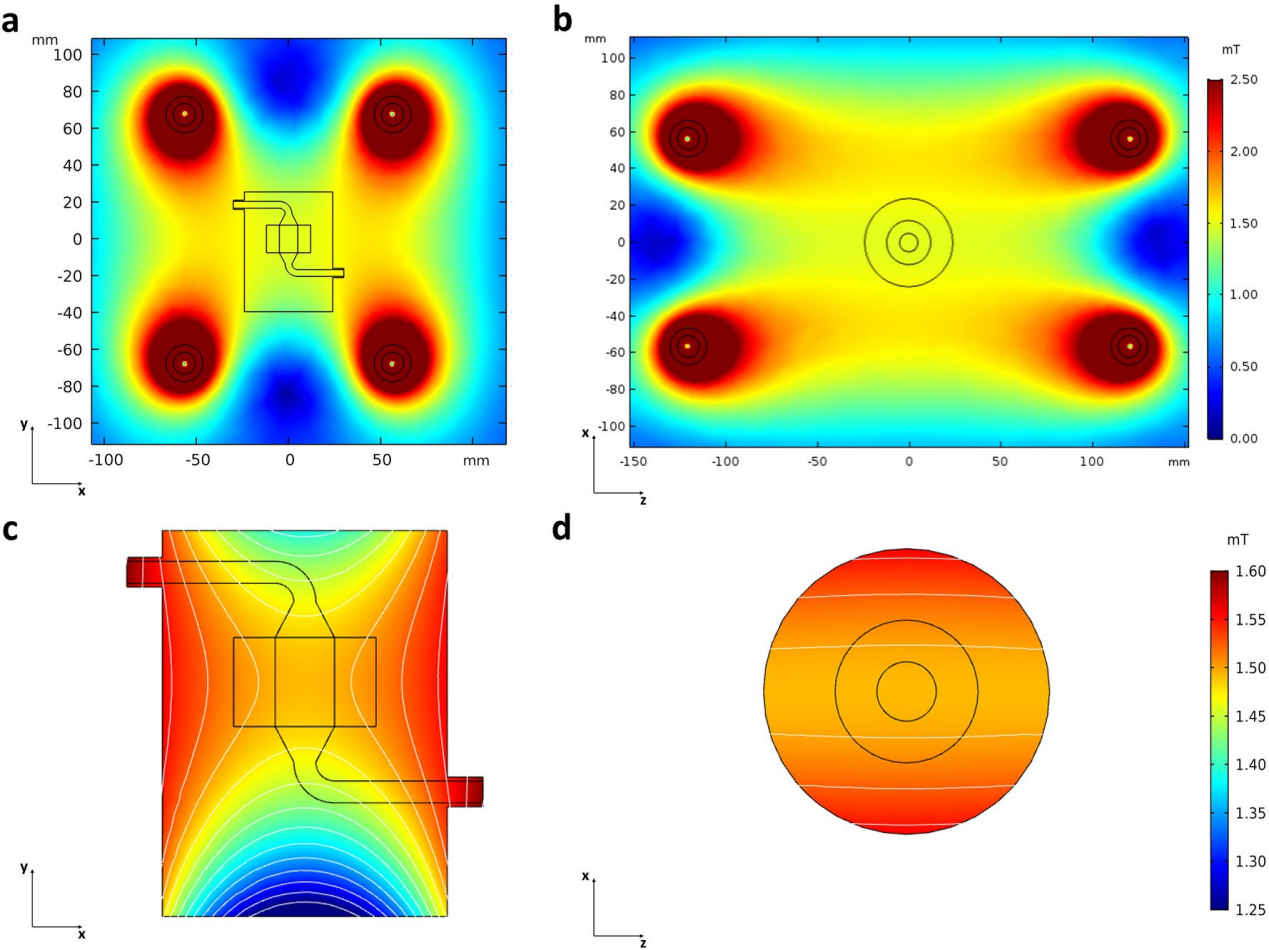
Contour plots with isolines of the magnetic field magnitude developing around and within the bioreactor culture chamber located between the PEMF stimulator solenoids. (**a**) Distribution of the magnetic field on the xy cross plane. (**b**) Distribution of the magnetic field on the xz cross plane. (**c**) Distribution of the magnetic field within the longitudinal section of the culture chamber. (**d**) Distribution of the magnetic field within the transverse section of the culture chamber.

The electromagnetic field simulations confirmed that positioning the bioreactor culture chamber between the two solenoids of the PEMF stimulator does not affect the magnetic field distribution, neither on the xy (Fig. 5a) or xz (Fig. 5b) cross planes. Additionally, the simulations clearly showed that the construct housed in the bioreactor culture chamber is exposed to a uniform magnetic field, as testified by the magnetic field magnitude isolines and the contour plots on the longitudinal (Fig. 5c) and transverse (Fig. 5d) sections of the culture chamber. The magnetic field intensity resulting on the region occupied by the construct is 1.5 mT, in accordance with the nominal value specified by the stimulator manufacturer.

### Assessments of 3D bone tissue models cultured under perfusion

**Figure 6:**
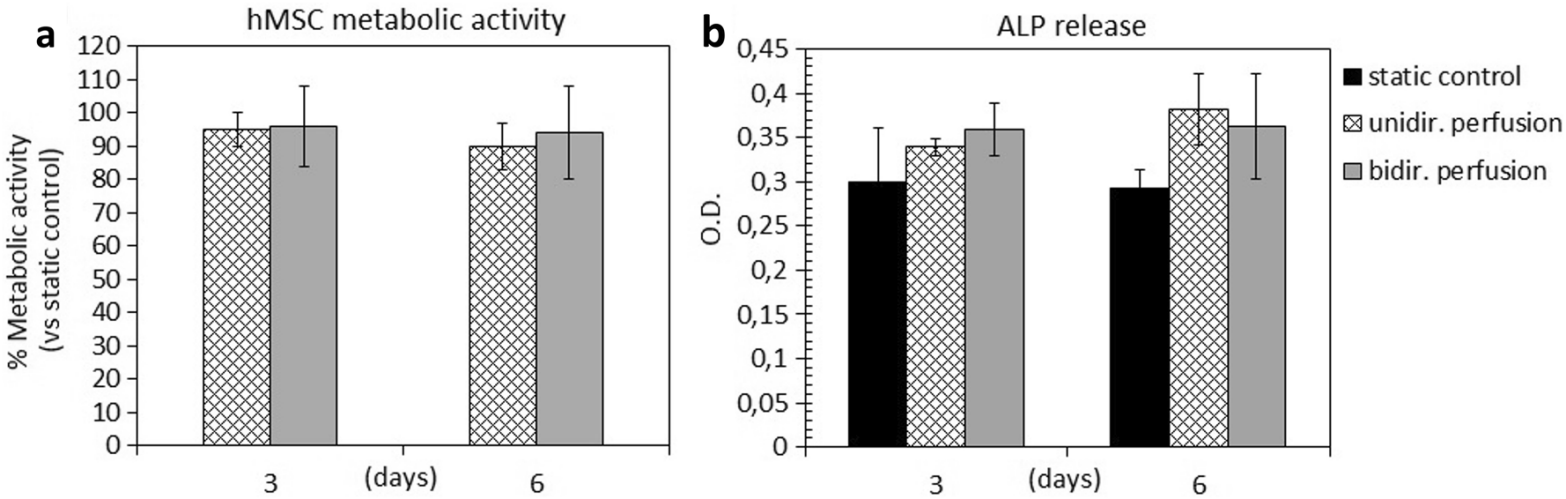
Biological assessments. (**a**) Metabolic activity of the 3D constructs cultured under uni-directional perfusion, and bi-directional perfusion assessed at day 3 and day 6; results are reported as % of the static control considered as 100% viability. (**b**) ALP released by the 3D constructs cultured under static conditions (control), uni-directional perfusion, and bi-directional perfusion assessed at day 3 and day 6 (p < 0.05 indicated by §). Bars represent means and standard deviations, replicates n = 3.

**Figure 7:**
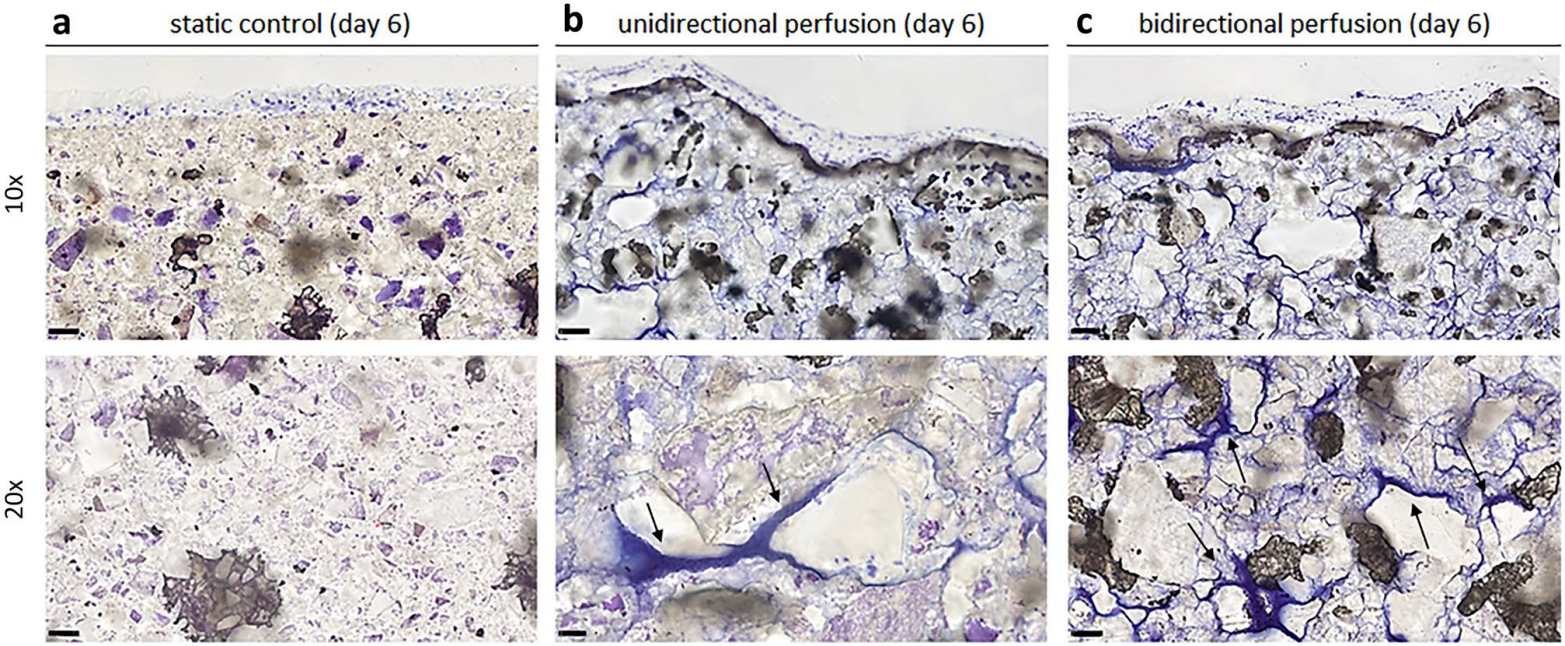
Histological images of the 3D constructs stained by Toluidine blue after 6 days of cultivation in (**a**) static conditions, (**b**) uni-directional perfusion, and (**c**) bi-directional perfusion. The black arrows indicate the ECM deposits throughout the constructs. 10 × magnification images, bar scale = 100 µm; 20 × magnification images, bar scale = 50 µm.

To assess the suitability of the bioreactor culture, 3D bone tissue models were cultured under uni- or bi-directional perfusion and in static conditions. The metabolic activity of cells exposed to uni- or bi-directional perfusion was comparable (> 90%, p > 0.05) to that of constructs cultured under static conditions, at both day 3 and day 6 (Fig. 6a). Interestingly and in accordance with literature, both uni- and bi-directional perfusion conditions determined an increase of the release of the early osteogenic marker ALP in the supernatant in comparison to static controls (p < 0.05, indicated by §, Fig. 6b). In particular, after 3 days of culture, an increase of almost 10–12% of ALP release was observed for the dynamic cultured constructs, and after 6 days of culture, the ALP release significantly increased up to 20–21%, as summarized in Table 1. No significant differences were observed by comparing the uni- and the bi-directional perfusion conditions (p > 0.05). As regard ECM deposition and distribution, the histological analysis of the constructs harvested at day 6 showed that both uni- and bi-directional perfusion conditions were effective in stimulating adherent cells to produce ECM in comparison to the static control (Fig. 7). In fact, the ECM deposits (stained in blue and indicated by arrows) were much more abundant in the perfused constructs (Fig. 7b,c) than in the static cultured ones (Fig. 7a), as it can be appreciated in both 10 × and 20 × magnification images. Moreover, comparing the uni- and the bi-directional perfusion conditions, a more homogeneous and consistent ECM deposition was observed for the bi-directional perfusion (Fig. 7c), demonstrating that such condition is more efficient in stimulating adherent cells to secrete ECM.

**Table 1:** Increase of ALP release under perfusion culture.

ALP release (% vs static control)		
Bioreactor setup	Day 3	Day 6
Uni-directional perfusion	+ 10.82%	+ 21.23%
Bi-directional perfusion	+ 12.23%	+ 20.72%

### Combined bi-directional perfusion and PEMF stimulation culture and Real-time PCR analysis

**Figure 8:**
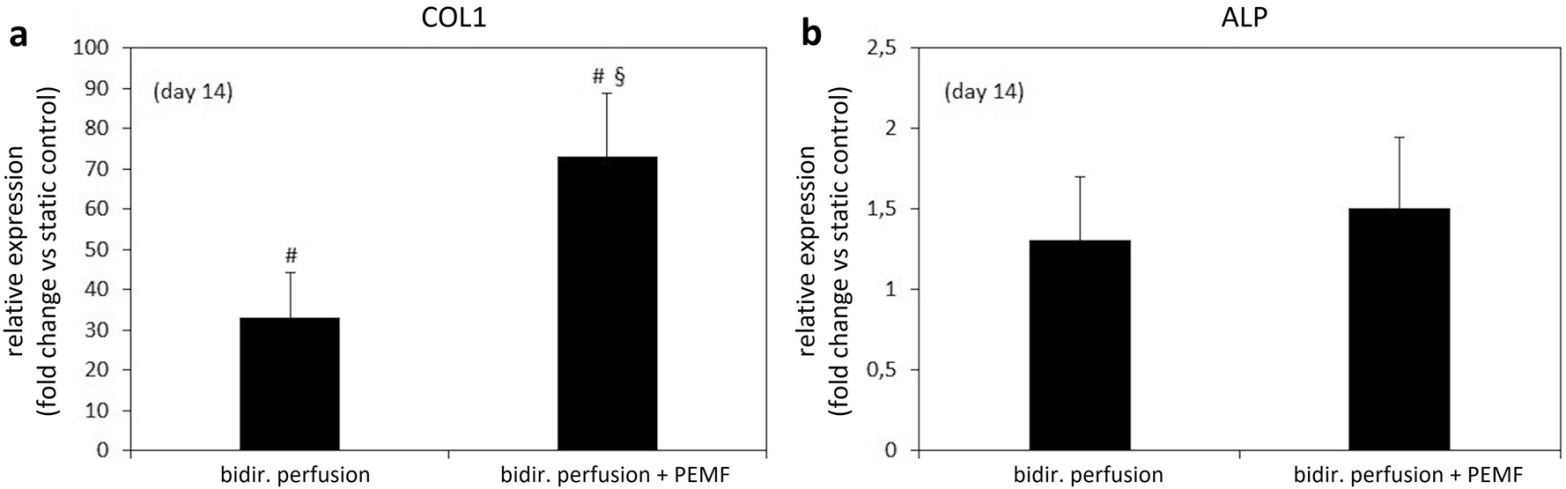
Real-time PCR results. (**a**) Collagen type I (COL1) and (**b**) alkaline phosphatase (ALP) genes expression of 3D bone tissue models cultured for 14 days under static culture (control), bi-directional perfusion, or bi-directional perfusion combined with PEMF stimulation (p < 0.05 with respect to control indicated by #, p < 0.05 with respect to bi-directional perfusion indicated by §). Bars represent means and standard deviations, replicates n = 3.

To verify the performances of the combined platform and to assess the biological effect induced by the combination of bi-directional perfusion and PEMF stimulation, 3D bone tissue models were cultured for 14 days under three defined conditions (static culture (control); bi-directional perfusion; bi-directional perfusion combined with PEMF stimulation) and using maintenance medium (DMEM) to avoid any biochemical osteogenic stimulus. At the end of the culture, the expression of the early ALP and late COL1 osteogenic genes was evaluated (Fig. 8). In particular, observing the expression of COL1 gene normalized to static culture (Fig. 8a), the bi-directional perfusion condition caused a ~ 40-fold increase in comparison to the control, confirming that the fluid flow-induced shear stress plays a crucial role in fostering the expression of collagen type I, even without biochemical stimulation. More interestingly, the combination of bi-directional perfusion and PEMF stimulation induced an even stronger pro-osteogenic effect, with an almost 80-fold increase in comparison to the control. This suggests that the further ~ 40-fold higher expression with respect to the bi-directional perfusion condition could be due to the effect of the secondary electric field induced in the constructs by the PEMF stimulation.

Less evident differences were observed on the expression of the early osteogenic marker ALP among the three culture conditions (Fig. 8b). In fact, the bi-directional perfusion condition led to a ~ onefold increase, whereas the combination of bi-directional perfusion and PEMF stimulation induced a ~ 1.5-fold increase with respect to the control.

## Discussion

In the emerging and multidisciplinary research field of mechanobiology, it is clearly recognized that physical stimuli arising from the surrounding microenvironment or externally applied play a crucial role in influencing cell fate^63,64^ and, at the tissue scale, tissue development, homeostasis, and even disease pathogenesis could be strictly dependent on physical forces^65^. In particular, bone and cartilage are among the tissues mostly influenced by mechanical stimuli in vivo: being deputed to the body support and mechanical stress dissipation, osteochondral tissues are highly exposed to compression and fluid flow-induced shear stress. In parallel, further physical stimuli, such as the non-invasive PEMF stimulation, are increasingly adopted in clinical practice for promoting endogenous bone healing^55^. However, a full understanding of the biological mechanisms induced in bone tissue by defined physical stimuli is still missing and the influence of different stimulation parameters and combinations is unknown^66^, leading to empirical treatments.

Inspired by the need of unrevealing how physical stimuli regulate cell and tissue functions, we developed a versatile automated perfusion bioreactor combinable with PEMF stimulation devices. As regards the bioreactor culture chamber, two layouts were designed (Fig. 1b,c) and compared in terms of hydrodynamic performances. The outcomes of the computational analysis confirmed that the internal geometry of the CC2 layout allows the development of a symmetric flow profile within the culture chamber and minimizes the regions with low or null longitudinal velocity (Figs. 3 and 4), reducing the risk of recirculation and stagnation zones. For these reasons and due to the limited working volume (2.5 mL), CC2 design was selected as the optimal geometry and was then manufactured by the SLA 3D-printing technique, which allowed fabricating the bioreactor following a single-step procedure, reducing complexity, costs and lead time^42,67–71^ (Fig. 2a). To provide controlled uni- or bi-directional perfusion, the closed-loop perfusion unit was equipped with a peristaltic pump suitable to be incubated and to be externally controlled. For the control unit, the selection of a low-cost open-source microcontroller board allowed combining control accuracy and reliability with compactness, flexibility, and cost-efficiency (Fig. 2b). Placed outside the incubator, the user-friendly control unit allows externally setting and automatically controlling the perfusion unit, while keeping constant the incubator conditions and reducing the contamination risk.

The automated perfusion control, which enables selecting uni- or bi-directional perfusion mode within the same platform and without user intervention along the culture, is the first significant advantage and novelty of the proposed bioreactor. Indeed, conventional bioreactors providing uni-directional perfusion are either non-automated^34,38^ or exploit automation strategies for regulating the flow rate, but do not allow reverting the flow direction^33^. On the other side, bioreactors delivering bi-directional perfusion are commonly based on syringe pumps^46,47,72^, which are unsuitable for providing continuous uni-directional perfusion. The only study that compared uni- and bi-directional perfusion on 3D bone constructs adopted two non-automated perfusion systems characterized by different total culture medium volumes (250 mL vs 1.5 mL, respectively)^48^. Differently, our bioreactor allows comparing uni- and bi-directional perfusion modes using the same device and total culture medium volume (50 mL).

As concerns the shear stress values induced by direct perfusion, several studies investigated the optimal range for in vitro dynamic culture of 3D bone tissue modes^20,73^. In detail, shear stress values ranging from 0.55 mPa to 24 mPa were shown to stimulate osteogenic differentiation of hMSCs and to promote ECM mineralization in both β-tricalcium phosphate^74^ and silk fibroin^75^ scaffolds. Differently, values exceeding 60 mPa were shown to result in cell death/detachment^76^, while values below 0.1 mPa did not stimulate any ECM mineralization^77^. In our study, the shear stress values developing within the construct under uni-directional perfusion were estimated to be 3.23 or 10.75 mPa, depending on the imposed flow rate (0.3 and 1.0 mL/min, respectively), thus confirming the suitability of the bioreactor for in vitro bone tissue culture and maturation.

As proof of concept, biological experiments imposing uni- or bi-directional perfusion mode on 3D bone tissue models, based on hMSCs seeded on Bio-Oss scaffolds, were performed. Being a pre-validated therapeutic product applied in clinic for small bone defects repair, Bio-Oss allowed specifically correlating the biological results with the applied perfusion conditions (uni- or bi-directional mode compared with the static control). To our knowledge, this is the first time that, thanks to automation, uni- and bi-directional perfusion modes were compared using the same platform and total culture medium volume. Firstly, it was demonstrated that the bioreactor perfusion culture does not introduce any disturbance for the cells in comparison to the static control in terms of metabolic activity (Fig. 6a). Actually, the imposed flow rate (0.3 mL/min) was appositely selected to mimic the interstitial fluid features in the native tissue^78^, providing native-like flow-induced shear stress to the cells. Moreover, no significant differences were observed between uni- and bi-directional perfusion conditions in terms of cell viability (Fig. 6a). The biological effect of the perfusion culture emerged from the evaluation of the early osteogenic marker ALP (Fig. 6b). In fact, just providing direct uni- or bi-directional perfusion for a short-term culture (6 days), without the use of osteogenic medium, a general increase of the ALP release was observed for the constructs cultured under perfusion compared with the static controls (+ 10–12% at day 3 and + 20–21% at day 6). This is in accordance with previous findings, showing that flow-induced shear stress alone is crucial for boosting osteogenic differentiation^48,79^. No significant differences were observed by comparing the uni- and the bi-directional perfusion modes in terms of ALP release; however, histological assessment enabled revealing the effect of the perfusion mode in terms of stimulation of cells in secreting and depositing ECM within the construct pores. Indeed, it is well known that flow-induced shear stress can influence ECM deposition^80,81^. In particular, in literature it was shown that uni-directional perfusion can induce an inhomogeneous ECM deposition within the construct, due to the different fluid transport and consequent shear stress values experienced by the cells located in the proximal and distal parts of the construct compared with the main fluid direction^48^. Conversely, bi-directional perfusion has been reported as an effective strategy to provide uniform mechanical stimulation over time to the construct, promoting osteogenesis in a more effective manner and inducing homogeneous ECM deposition along the whole 3D bone tissue models^48^. In our study, the histological images of the 3D constructs harvested after 6 days of uni- or bi-directional perfusion and stained with Toluidine blue confirmed that perfusion favors cells to better penetrate the scaffold structure as well as to deposit more ECM compared with static conditions. Notably, the ECM deposition was more marked for the constructs exposed to bi-directional perfusion, confirming the hypothesis that a bi-directional flow can represent a more efficient culture condition for 3D constructs mimicking physiological tissues. In particular, Toluidine blue was selected for the staining of the 3D bone tissue models as it specifically visualize the proteoglycan content in a tissue^82^. Proteoglycans and glycoproteins represent the majority of the non-collagenous proteins of the bone matrix. During the bone healing process, proteoglycans such as decorin, biglycan, and osteoadherin play a pivotal role in promoting and supporting the early mineralization of the matrix in the first stages of osteogenesis. Therefore, it can be speculated that the regions positive to the Toluidine blue staining showing a dark-blue pigmentation are representative of early mineralization occurring in the pores of the constructs cultured under perfusion. These experiments were performed for a short culture time (6 days) to assess the suitability of the developed bioreactor to culture 3D bone tissue models under a physiological condition such as perfusion. Moreover, the analysis of the ALP release and the histology of the deposited ECM allowed determining the optimal perfusion mode (i.e., bi-directional perfusion) to be applied for further tests dealing with the investigation of the combined stimulation (bi-directional perfusion + PEMF stimulation).

Indeed, the results from the electromagnetic field modelling showed that the proposed bioreactor is in principle suitable to be combined with a commercial PEMF stimulator for exposing 3D bone tissue models to uni-/bi-directional flow-induced shear stress and uniform pulsed electromagnetic field (Fig. 5). Based on the preliminary biological results, dedicated biological tests were then performed for verifying the performances of the combined platform and for investigating the potential pro-osteogenic effect on 3D bone tissue models of combining bi-directional perfusion and PEMF stimulation. The constructs were cultured for 14 days under three different conditions (i.e., static culture (control), bi-directional perfusion, and bi-directional perfusion combined with PEMF stimulation) and, for all experiments, the maintenance medium (DMEM) was adopted for avoiding the interference of biochemical stimulation that could lead to misleading interpretation of the effects of the applied physical stimulations. At day 14, the expression of the early osteogenic gene ALP and the late osteogenic gene COL1 were evaluated by Real-time PCR (Fig. 8) to provide a quantitative evaluation of the effect of individual or combined applied stimuli in terms of pro-osteogenic boost. Results revealed that bi-directional perfusion per se induced a ~ 40-fold up-regulation of COL1 in comparison to the control, and the further combination with PEMF stimulation boosted the up-regulation of COL1 up to ~ 80-fold (Fig. 8a). This result represents a promising evidence that the combination of bi-directional perfusion and PEMF stimulation leads to a positive synergic contribution in promoting the expression of COL1, which is a fundamental component of the bone matrix. Indeed, collagen type I represents the 90–95% of the organic components of the bone tissue and is one of the key factors in determining the bone mechanical properties (particularly elasticity and flexibility)^83^. At the cellular and subcellular level, this synergic effect is due to the combination of the fluid flow-induced shear stress and the PEMF secondary electric field induced in the constructs. Fluid flow-induced shear stress acts on the cell membrane and can deform it, leading to alteration of membrane proteins and causing mechano-activated ion channels to open and allow the influx of cations, such as Ca^2+^, Na^+^, and K^+^, into the cell^36,84^. In parallel, PEMF stimulation can trigger the depolarization of the cell membrane and consequently stimulate ion currents, such as opening the Ca^2+^ channels and leading to an intracellular Ca^2+^ ion accumulation^85,86^. Such ions unbalance can activate specific cascades, such as the nuclear factors of activated cells (NFAT), enabling the transcription and the synthesis of osteogenic proteins^87^. Regarding the less evident effect of up-regulation observed for the ALP expression, although present (Fig. 8b), it should be noted that several studies reported ALP to be upregulated within 2 days of osteogenic induction^88^; therefore, 14 days could represent a too long culture time for appreciating the effect of physical stimulation on ALP expression.

In literature, only the study of Wang and colleagues combined a perfusion bioreactor with a sinusoidal electromagnetic field (EMF) generator^52^. In detail, rabbit MSCs were seeded on hydroxyapatite/collagen scaffolds and the constructs were cultured for 14 days under uni-directional perfusion (10 mL/min) and EMF stimulation (magnetic field intensity = 1 mT, frequency = 15 Hz, exposure time = 4 h/day) with and without osteogenic culture medium, obtaining enhanced osteogenic differentiation at the end of the culture, similarly to our results. However, the system proposed by Wang et al. allowed delivering only uni-directional perfusion with no automated control of the pump^52^. Therefore, a further advantage and novelty of the here presented platform is related to its versatility to combine automated uni-/bi-directional perfusion and PEMF stimulation, which was demonstrated to be crucial for boosting pro-osteogenic differentiation in 3D bone tissue models.

Some limitations could affect this study. In the CFD modelling the construct was assumed as a homogeneous and isotropic porous medium, lacking the information about the real microarchitecture of the Bio-Oss scaffold and neglecting the presence of the cells. Moreover, the simulations did not take into account that along the culture the construct geometry is modified by cell proliferation and ECM deposition, which cause a decrease of the mean pore size and an increase of shear stress values over time. For these reasons, the computed shear stress values can be considered as a reasonable estimation for the early culture stage and in the future micro-computed tomography imaging of the constructs will be performed at day 0 and at different time points in order to precisely characterize the flow dynamics within the construct along its maturation^89–91^. As regards the electromagnetic field modelling, it was performed in steady-state conditions. Although this approach neglects the temporal evolution of the magnetic field occurring during a PEMF pulse, the results were sufficiently accurate for describing the conditions occurring at the pulse peak while allowing a significant reduction of the computational costs. Concerning the biological tests, it should be noted that for the perfusion condition an higher culture medium volume was used compared with the static condition (50 mL vs 3 mL changed every 3 days, respectively). A higher culture medium volume provides a higher amount of nutrients, and it could be speculated that this aspect, rather than fluid flow-induced shear stress, could favor the metabolic activity of the cells cultivated under perfusion compared with the statically cultured ones. However, the fact that the ECM deposition was more marked for the constructs exposed to bi-directional perfusion compared with the ones cultured under uni-directional perfusion (same culture medium volume) confirms that perfusion plays a beneficial role in ECM deposition. Lastly, the adopted Bio-Oss scaffold probably further contributed to stimulate osteogenesis due to its chemical composition. However, since all the constructs cultured under either static or dynamic conditions were based on Bio-Oss scaffolds and were cultured using the same basal medium (DMEM), the observed differences can be directly ascribed to the applied culture conditions.

## Conclusion

In this study, we developed, characterized, and tested a tunable perfusion bioreactor based on automated control that can be combined with PEMF stimulation devices to be used as powerful tool for in vitro BTE production and investigations. The bioreactor is highly versatile as it allows housing constructs of different size and delivering individual or combined flow-induced shear stress and PEMF stimulations. Moreover, the adopted automation strategy enables providing uni- or bi-directional perfusion within the same platform and using the same total culture medium volume, significantly reducing the user intervention and dependence along the culture and increasing robustness and reproducibility of the culture process. The preliminary biological tests on perfusion demonstrated that the only application of perfusion was crucial for promoting osteogenic differentiation in the cultured constructs, even without the use of biochemical stimulation. In fact, uni- and bi-directional perfusion conditions were effective in stimulating the osteogenic differentiation of the cultured 3D bone tissue models, and highlighted that bi-directional perfusion better promoted the ECM deposition throughout the construct. Lastly, as regards PEMF stimulation, biological results demonstrated the synergic pro-osteogenic effect of combining bi-directional perfusion and PEMF stimulation and confirmed that the proposed platform could be used for both the production of BTE constructs and as powerful investigation tool. In the next future, an advanced investigation approach, based on the proposed bioreactor and high-throughput analyses, could lead to unravel molecular mechanisms activated by biophysical stimulation applied in clinic and to define the precise combinations of parameters inducing specific biological effects, paving the way for optimized orthopedic clinical protocols.

## Supplementary Information

Supplementary Information.

## Data Availability

The raw data supporting the conclusions of this article will be made available by the corresponding author, without undue reservation, to any qualified researcher.
