# Supplementary material for: An automated 3D-printed perfusion bioreactor combinable with pulsed electromagnetic field stimulators for bone tissue investigations
Source: Sci Rep. 2022 Aug 16;12:13859. doi: 10.1038/s41598-022-18075-1 (PMC9381575; doi:10.1038/s41598-022-18075-1)
Supplement: Supplementary file 1 — Supplementary Information. [file 41598_2022_18075_MOESM1_ESM.pdf]

Supplementary Material

Preliminary performance tests

The watertightness and reliability of the bioreactor were preliminarily tested in-house.
Firstly, the CC2 underwent 5 autoclave cycles, to assess the maintenance of geometry and functionality. No deformations were observed in the CC2 components.
Secondly, the CC2 was connected to the perfusion unit and tested in uni-directional perfusion mode with and without a reference scaffold (Bio-Oss, Geistlich Pharma AG, Switzerland), using distilled water at room temperature and imposing the highest flow rate provided by the pump (24 mL/min) for 58 h. No leakage was observed both with and without the reference scaffold inserted. Moreover, in the case of scaffold inserted, the applied bottom-to-top uni-directional perfusion efficiently promoted the outflow of air, preventing the residence of air bubbles that could impair the culture process.
Lastly, the control unit was connected to the pump and the bi-directional perfusion mode was tested setting different cycle durations and checking the inversion timing using a stopwatch. The control unit timing respected the prescribed conditions.

Supplementary Table S1. Perfusion parameters

Parameter	Range
Tubing size (internal diameter)	1.0 or 2.4 mm
Flow rate	0.006 - 6 mL/min (1.0 mm tubing)<br>0.024 - 24 mL/min (2.4 mm tubing)
Perfusion mode	uni-directional/bi-directional
Direction of rotation	clockwise/counterclockwise
Cycle duration	1 s - 24 hours

Supplementary Table S2. Electromagnetic properties of the modelled domains (when the properties were unknown, an analogous material was considered).

Domain	Electric conductivity (S/m)	Relative permittivity	Relative permeability
Culture chamber (photopolymer)	$1*10^{-14}$	3.1	1
Holder (PDMS)	$2.5*10^{-14}$	2.4	1
Construct (glass ceramic) [1]	$1*10^{-7}$	24.5	1
Culture medium [2,3]	1.45	80	1
Coils (copper)	$5.998*10^{7}$	1	1
Coils' cover (PVC) [4]	$1.33*10^{-13}$	4.46	1
Air	0	1	1

1. Pisitpipathsin, N. *et al.* Effect of BCZT on Electrical Properties and Bioactivity of 45S5 Bioglass. *Integrated Ferroelectrics* 142, 144–153 (2013).

2. Chen, M.-T., Jiang, C., Vernier, P. T., Wu, Y.-H. & Gundersen, M. A. Two-dimensional nanosecond electric field mapping based on cell electropermeabilization. *PMC Biophys* 2, 9 (2009).

3. Tandon, N. *et al.* Optimization of electrical stimulation parameters for cardiac tissue engineering. *J Tissue Eng Regen Med* 5, e115–e125 (2011).

4. Mukherjee, A. K. & Patri, M. Electrical properties of chlorinated atactic polypropylene (CAPP) and PVC–DOP–CAPP system. *Journal of Applied Polymer Science* 39, 1485–1493 (1990).

Supplementary Table S3. Mean velocity and wall shear stress values within the construct calculated for the different modelled conditions.

Chamber layout	Flow rate (mL/min)	Mean velocity within the construct (m/s)	Wall shear stress (mPa)
CC1	0.3	6.37×10^{-5}	3.23
	1	2.12×10^{-4}	10.75
CC2	0.3	6.37×10^{-5}	3.23
	1	2.12×10^{-4}	10.75

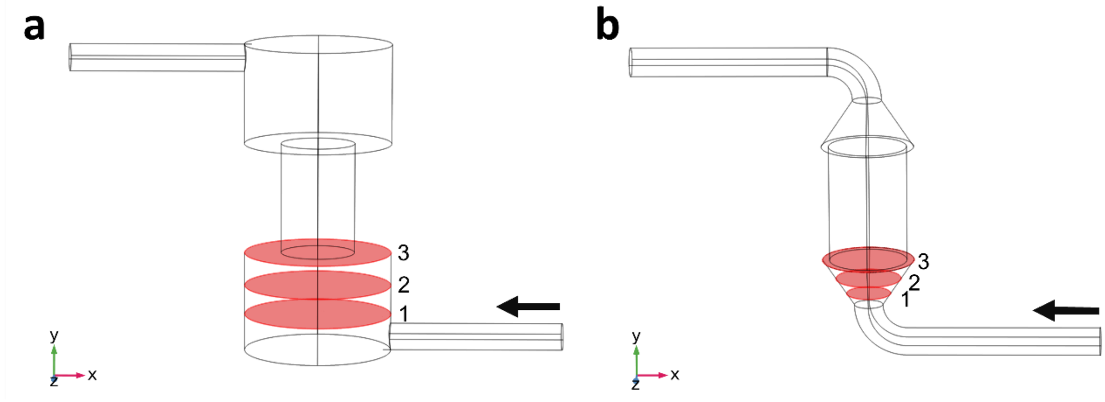

Supplementary Figure S1. 3D view of the internal geometry of CC1 (a) and CC2 (b), highlighting the direction of flow perfusion and the 3 horizontal sections where the fluid velocity field distributions were analyzed: 1) at 0.5 mm above the end of the inlet channel; 2) at midway between the construct and the end of the inlet channel (i.e., 4.5 mm below the construct for CC1 and 2.5 mm below the construct for CC2); 3) tangent to the construct.

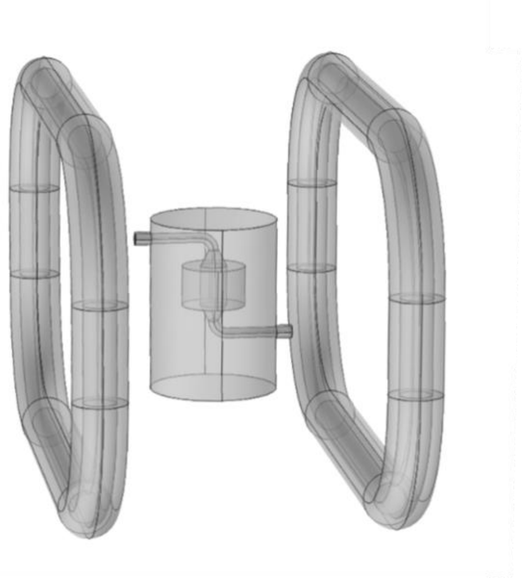

Supplementary Figure S2. 3D geometry considered for the stationary electromagnetic field modelling.

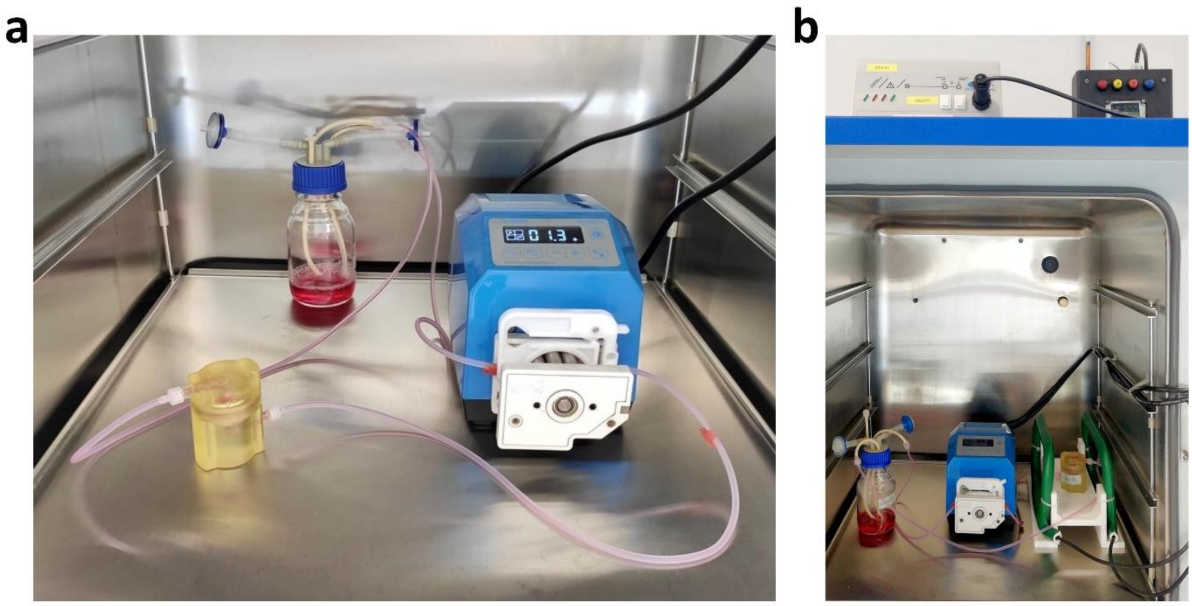

Supplementary Figure S3. (a) Picture of the bioreactor setup within the incubator, with CC2 connected by silicone tubing to the reservoir and the peristaltic pump. This latter is connected by electric wires to the control unit located outside the incubator. (b) Picture of the bioreactor setup combined with the PEMF stimulator. Within the incubator, CC2 is placed between the PEMF stimulator coils and connected by silicone tubing to the reservoir and the peristaltic pump. The coils are connected by electric wires to the PEMF stimulator located outside the incubator. Similarly, the pump is connected to the control unit located outside the incubator.

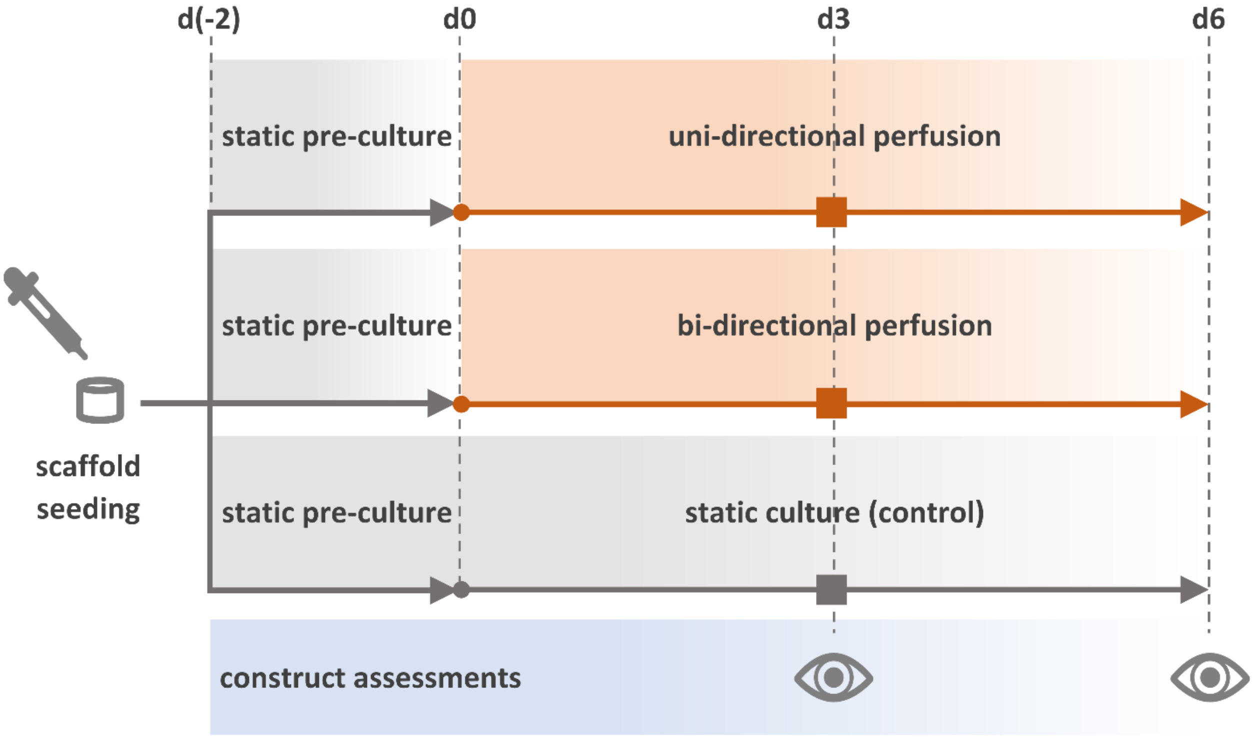

Supplementary Figure S4. Timeline of the performed culture protocols.

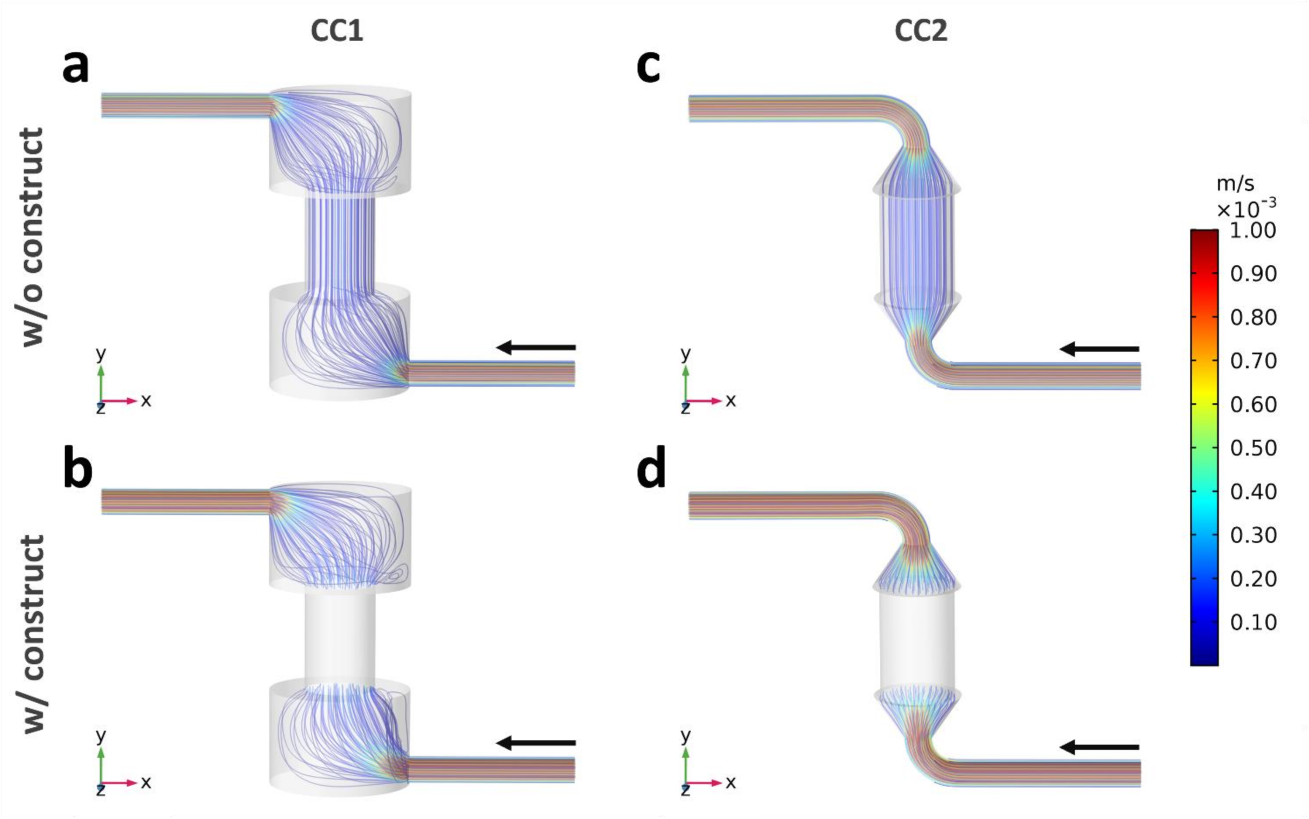

Supplementary Figure S5. Flow streamlines developing within CC1 and CC2 imposing a modeled flow rate of 1 mL/min and color-coded with respect to velocity values. (a) CC1 without construct. (b) CC1 with an inserted construct modelled as porous medium. (c) CC2 without construct. (d) CC2 with an inserted construct modelled as porous medium.
